# *Bifidobacterium longum* CECT 7347 Modulates Immune Responses in a Gliadin-Induced Enteropathy Animal Model

**DOI:** 10.1371/journal.pone.0030744

**Published:** 2012-02-10

**Authors:** José Moisés Laparra, Marta Olivares, Onofrio Gallina, Yolanda Sanz

**Affiliations:** Institute of Agrochemistry and Food Technology, National Research Council (IATA-CSIC), Valencia, Spain; French National Centre for Scientific Research - Université Aix-Marseille, France

## Abstract

Coeliac disease (CD) is an autoimmune disorder triggered by gluten proteins (gliadin) that involves innate and adaptive immunity. In this study, we hypothesise that the administration of *Bifidobacterium longum* CECT 7347, previously selected for reducing gliadin immunotoxic effects *in vitro*, could exert protective effects in an animal model of gliadin-induced enteropathy. The effects of this bacterium were evaluated in newborn rats fed gliadin alone or sensitised with interferon (IFN)-γ and fed gliadin. Jejunal tissue sections were collected for histological, NFκB mRNA expression and cytokine production analyses. Leukocyte populations and T-cell subsets were analysed in peripheral blood samples. The possible translocation of the bacterium to different organs was determined by plate counting and the composition of the colonic microbiota was quantified by real-time PCR. Feeding gliadin alone reduced enterocyte height and peripheral CD4+ cells, but increased CD4+/Foxp3+ T and CD8+ cells, while the simultaneous administration of *B. longum* CECT 7347 exerted opposite effects. Animals sensitised with IFN-γ and fed gliadin showed high cellular infiltration, reduced villi width and enterocyte height. Sensitised animals also exhibited increased NFκB mRNA expression and TNF-α production in tissue sections. *B. longum* CECT 7347 administration increased NFκB expression and IL-10, but reduced TNF-α, production in the enteropathy model. In sensitised gliadin-fed animals, CD4+, CD4+/Foxp3+ and CD8+ T cells increased, whereas the administration of *B. longum* CECT 7347 reduced CD4+ and CD4+/Foxp3+ cell populations and increased CD8+ T cell populations. The bifidobacterial strain administered represented between 75–95% of the total bifidobacteria isolated from all treated groups, and translocation to organs was not detected. These findings indicate that *B. longum* attenuates the production of inflammatory cytokines and the CD4+ T-cell mediated immune response in an animal model of gliadin-induced enteropathy.

## Introduction

Coeliac disease (CD) is an autoimmune enteropathy triggered by cereal gluten proteins (gliadin) in genetically predisposed individuals [1]. In CD patients, peptides resulting from incomplete protein hydrolysis by digestive enzymes cause a deregulated immune response and inflammation. The degree of intestinal inflammation can vary from intraepithelial lymphocytosis to severe infiltration of mononuclear cells in the lamina propria, causing villous atrophy and crypt cell hyperplasia in the small intestine [2].

Several attempts have been made to develop animal models that reproduce CD pathogenesis, including the immune response, the mucosal lesions and the symptoms [3]–[5]. The intragastric administration of gliadin to inbred rats after weaning [4] or to immunocompetent mice at 10 days of age failed to reproduce the damage of the intestinal mucosa [6]. Human leukocyte antigen (HLA)-DQ8/HCD4 or single HLA-DQ8 transgenic mice sensitised with gluten developed an immune response to gliadin that involved both the adaptive and innate immune system [7], [8] and dependent changes in gut neuromuscular and epithelial secretory function [8], but did not develop a gluten-dependent enteropathy. Nevertheless, repeated oral administration of gliadin to rats, previously sensitised with interferon gamma (IFN-γ) immediately after birth, caused mucosal lesions characterised by shortening of jejunal villi, crypt hyperplasia, and increased cellular infiltration, including CD8αβ+ and CD4+ T lymphocytes [9]. Activation of CD4+ T-helper 1 (Th1) cells that produce IFN-γ and intraepithelial CD8+ lymphocytes are also responsible for the cytotoxic effects on intestinal epithelium, which in turn could increase passage of gliadin antigens to the lamina propria and further activate the CD4+ Th1 cell response [10], [11]. Thus, this model reproduces a CD4+ T cell mediated enteropathy, defined as hyperplasic-infiltrative (type II), similar to that described in CD patients [9]. Undoubtedly, further refinement of the available animal model of CD is desirable, but it is considered appropriate to initially explore pathogenic mechanisms and potential pharmaceutical or nutritional interventions [9].

The production of T cells with regulatory activity (Tregs) constitutes one of immunosuppressive mechanisms that contribute to intestinal tolerance and prevention of autoimmunity. In particular, natural self-antigen-reactive CD4+CD25+ cells acquired Foxp3 expression, a key marker of the development of regulatory activity, in the thymus and then enter peripheral tissues, where they can suppress the activation of other self-reactive T cells contributing to immune tolerance. These Tregs (CD4+CD25+Foxp3+) are particularly increased in the mucosa and peripheral blood of active CD patients as a consequence of the activation of a regulatory response to counteract the inflammation caused by gluten [12], [13], but their role in animal models of CD has not been studied so far.

In recent years, innate immunity and early interactions of gliadin-derived peptides with intestinal epithelial cells have also been considered critical in the development of the disease. Some gluten peptides can mediate an innate-immune response that involves induction of interleukine (IL)-15 production by epithelial and dendritic cells. IL-15 induces up-regulation of the non-MHC class I receptor NKG2D on intraepithelial lymphocytes (IELs), and its ligand MICA on epithelial cells, that interact and activate cytolytic function on enterocytes [14]. The activation of the NFκB pathway in intestinal epithelial cells also mediates the production of other inflammatory cytokines, such as the tumour necrosis factor (TNF)-α, which facilitates the interaction of IELs and intestinal epithelial cells promoting tissue inflammation [15].

In germ-free rat pups, colonisation by the whole microbiota has similar effects as administration of gliadin on IEL subpopulations, suggesting that both factors activate common immunological responses that may influence CD development [4]. Human studies also report that CD is characterised by imbalances in the composition of the microbiota and, particularly, reduced numbers of total bifidobacteria and *B. longum* [16]. *In vitro* studies have demonstrated that the presence of *B. longum* CECT 7347 during the intestinal digestion of gliadin leads to the generation of different peptide sequences and reduces their toxic and inflammatory effects on intestinal epithelial cells [17]. In addition, *B. longum* CECT 7347 has been shown *in vitro* to counteract the inflammatory response induced by the altered faecal microbiota of CD patients in peripheral blood mononuclear cells [18]. Yet, the possible *in vivo* effects of this bifidobacterial strain on CD have not been evaluated.

In the light of the evidence available, in this study we hypothesise that the administration of *B. longum* CECT 7347, with immunoregulatory properties and ability to attenuate *in vitro* gliadin toxicity on epithelial cells, could exert protective effects in a model of gliadin-induced enteropathy in weaning rats.

## Materials and Methods

### Bacterial strain and culture conditions

*Bifidobacterium longum* CECT 7347 was isolated from faeces of healthy infants as described elsewhere [18]. The bacterial cultures were grown in Man-Rogosa-Sharpe agar and broth (Scharlau, Barcelona, Spain) supplemented with 0.05% (w/v) cysteine (MRS-C; Sigma-Aldrich, St. Louis, USA), and kept at 37°C in anaerobic conditions (AnaeroGen, Oxoid, Basingstoke, UK) for 24 h. For animal studies, a pure culture of the strain was grown overnight and used to inoculate fresh MRS-C broth for 22 h. Cells were harvested by centrifugation (6,000×g for 15 min) at stationary growth phase, washed twice in phosphate buffered saline (PBS, 130 mM sodium chloride and 10 mM sodium phosphate, pH 7.4), and re-suspended in 10% (w/v) hypoallergenic milk-based formula (Nutramigen©, Mead Johnson B.V., Nijmegen, Netherlands). Aliquots of these cell suspensions were frozen in liquid nitrogen and stored at −80°C until used. The number of live cells after freezing and thawing was determined by plate counting on MRS-C agar after 48 h of incubation, and were expressed as colony-forming units (CFU) per mL. More than 90% of cells were alive upon thawing and no significant differences were found during storage time (4 months). One fresh aliquot was thawed for every new experiment to avoid variability in bacterial cell viability between experiments.

### Animals and experimental design

Animal experiments were carried out in strict accordance with the recommendations in the Guide for the Care and Use of Laboratory Animals of University of Valencia (SCSIE, University of Valencia, Spain) and the protocol was approved by its Ethic Committee. Experimental animals were female, weaning Wistar rats, provided by the SCSIE. The adult females were date-mated, and fed *ad libitum* with a standard diet (Harlan Bioproducts, Indianapolis, USA). Shortly after spontaneous birth, animals were randomly distributed into seven different groups (n = 6 per group): 1) artificially reared (AR) with the hypoallergenic milk-based formula; 2) AR and fed *B. longum* CECT 7347; 3) AR and fed gliadin-derived peptides (GP); 4) AR and fed GP and *B. longum* CECT 7347; 5) AR sensitised with 1,000 U IFN-γ administered intraperitoneally immediately after birth; 6) AR sensitised with 1,000 U IFN-γ administered intraperitoneally immediately after birth and fed GP; 7) AR sensitised with IFN-γ and fed GP and *B. longum* CECT 7347.

Newborn animals were hand-fed (100 µL) using a micropipette every 4 hours (4–5 times a day) until the age of 10 days. They were fed with the hypoallergenic milk-based formula for newborns, composed of: 19 g proteins; 34 g fats; 75 g carbohydrates; 680 Kcal; 260 mOsm per L. The bacterium was administered at a concentration of 6.0×10^7^–8.2×10^8^ CFU/day, as determined by plate counting on MRS-C agar, in a single dose during the 10 days. To obtain the GP, a commercially available extract of gliadin (Sigma-Aldrich, St. Louis, USA) was submitted to *in vitro* digestion and then dialysed using a 15 kDa cut-off membrane [17]. Weaning rats were fed 50 µg gliadin/day in a single dose during the 10 days, and, finally, they received a provocative dose of gliadin 100 µg ∼2 hours before sacrifice.

Changes in body weight were monitored every two days. After treatment, rats were anaesthetised (isofluran) and killed by exsanguination. Whole blood samples were preserved in EDTA-treated tubes to prevent coagulation (at room temperature) for leukocyte analyses. Sections (1 cm) of the proximal jejunum were immersed in RNA *later* buffer (Qiagen, USA) or Krebs's buffer and kept at −80°C for gene expression and cytokine analyses. Liver, spleen and colon content samples were also collected in PBS and immediately used for microbiological analyses by plate culturing.

### Histologic and morphometric evaluation

Jejunal tissue sections (1 cm) were fixed in formaldehyde 10% (p/v) and then sections of 5 µm were stained with haematoxylin-eosin staining. The samples were analysed with a Nikon Eclipse 90i microscope equipped with a Nikon DS-5Mc digital camera. Photos were analysed with the Nis Elements software (Nikon Instruments Inc., Melville, USA). The parameters analysed included, villi width and length and number of infiltrated cells in the lamina propria, because their changes characterized the histologic lesions of this enteropathy, and the number and height of enterocytes that provide an indication of the disorganization of the cellular epithelial layer.

### Leukocyte counts

The morphological identification of immune cells was conducted by the May-Grünwald Giemsa's staining procedure. Aliquots (25 µL) of blood samples were extended on glass slides and were allowed to air-dry. The samples were covered with May-Grünwald's solution (Sigma-Aldrich, St. Louis, USA) for 3 minutes, and afterwards an equal volume of phosphate buffered distilled water (PBDW, pH 7) was added and incubated for 3 additional minutes. The preparations were gently rinsed with PBDW, and then covered with a dilution (1∶20, v/v) of the Giemsa stain-modified solution in PBDW for 12 minutes. Finally, the samples were washed with PBDW, air-dried and analysed using an Olympus BX51 microscope (Madrid, Spain).

### Lymphocyte phenotyping

Aliquots (100 µL) of peripheral blood were mixed with the following fluorochrome-conjugated antibodies: Anti-rat CD45, CD4, CD8 and Foxp3 (eBiosciences, Hattfield, UK). Then samples were prepared for flow cytometry analysis with the Immunoprep kit (Beckman Coulter, USA) according to the manufacturer's instructions, and further analysed on a Modular Flow Cytometer Cell Sorter (MoFlo Sorter, Dakocytomation, USA).

### Real-time reverse transcription-polymerase chain reaction (RT-qPCR)

Total RNA was extracted from tissue samples with the RNeasy mini kit (Qiagen) according to the manufacturer's instructions. One microgram of total RNA was converted to double-stranded cDNA using AMV Reverse Transcriptase (Promega, Madison, USA). PCR was performed with primers designed for the following *Rattus norvegicus* genes: NFκB (forward 5′- CTT CTC GGA GTC CCT CAC TG-3′, reverse 5′- CCA ATA GCA GCT GGA AAA GC-3′) and β-actin (forward 5′- CTC TTC CAG CCT TCC TTC CT-3′; reverse 5′- TAG AGC CAC CAA TCC ACA CA-3′), the latter used as a housekeeping gene. The PCR mix (20 µL reaction volume) consisted of 7.5 µL SYBR Green I master mix, 1.3 µmol/L primers, and 2.5 µL cDNA. PCR reactions were performed in triplicate in a LightCycler® 480 (Roche) system with the following conditions: 1 cycle at 95°C for 5 min, 35 cycles at 60°C for 20 s and 72°C for 45 s. The relative mRNA expression of the tested gene relative to β-actin expression was calculated using the 2^−ΔΔCp^ method [19]. Samples of each animal tissue were measured in duplicate and gene expression was expressed as fold-change.

### Cytokine protein assay

Jejunal tissue sections (1 cm) were kept in Krebs's buffer (1 ml) supplemented with protease inhibitors (Roche) until analysis. Tumour necrosis factor-α (TNF-α, Diaclone, Besançon, France), and interleukine (IL)-10 (Abcam, Cambridge, UK) were determined by ELISAs according to the manufacturer's instructions. Prior to cytokine determination, tissue samples were homogenised in cell lysis buffer using a TissueRuptor (Qiagen). Then, samples were centrifuged (1000×g, 15 min) to get clear supernatants for cytokine determination. The results of the ELISA assay are expressed as pico-grams per gram of tissue (pg/g).

### Microbiological analyses

Aliquots (50 mg) of different biologic samples (colon content, mesenteric lymph nodes [MLN], spleen and liver) were diluted (1/9) in PBS and decimal dilutions were plated on MRS-C agar supplemented with mupirocin (80 mg/L) (Sigma-Aldrich) and acetic acid (1 ml/L) to increase the selectivity of the medium for bifidobacteria. Counts were performed on the highest dilution plates and were expressed as colony forming units (CFU) per gram of faeces. Isolated colonies (8–10) from colon samples were also checked by RAPD PCR analysis to confirm whether the DNA profile of the isolates corresponded with the DNA profile of a pure culture of the administered strain *B. longum* CECT 7347. The random primer 1254 (5′-CCG CAG CCA A-3′) was used for RAPID-PCR analysis as previously described [20]. The RAPD-PCR products were visualised on a 1.5% w/v agarose gel after staining with ethidium bromide.

The composition of the microbiota was also analysed by real-time PCR. Samples of colon content were collected, diluted 1∶10 (w/v) in PBS (pH 7.2) and homogenised thoroughly by agitation in a vortex. Aliquots were used for DNA extraction using the QIAamp DNA stool Mini kit (Qiagen, Hilden, Germany) following the manufacturer's instructions. Genus-, group- and species-specific primers were used as described previously to quantify the different bacterial groups of the intestinal microbiota [21], [22]. Briefly, PCR amplification and detection were performed with an ABI PRISM 7000-PCR sequence detection system (Applied Biosystems, UK). Each reaction mixture (15 µl) consisted of 7.5 µl of SYBR® Green PCR Master Mix (Roche), 3.5 µl of sterile water, 0.75 µl of each of the specific primers at a concentration of 10 µM, and 2.5 µl of template DNA. 16 rRNA gene copy numbers of each bacterial group or species were calculated by comparing the C_t_ values obtained with those from a standard curve [23]. Standard curves were generated from serial dilutions of a known copy number of the target gene cloned into a plasmid vector. For each reference strain the 16S rRNA gene was cloned into a pGEM-T Easy Vector System (Promega). An *E. coli* strain was transformed with the recombinant plasmids and plasmid DNA was extracted from *E. coli* by the miniprep method [24]. Six non-zero standard concentrations were used to construct the standard curves for each reference strain representing a species or a group, and the plasmid DNA concentrations ranged from 10^4^ to 10^10^ copies of DNA per reaction. Standard curves were constructed by plotting the C_t_ values against the logarithm of their initial template copy number. DNA concentration was measured using a NanoDrop® and the corresponding copy number was calculated [25]. The following reference strains were used as standards: *Bifidobacterium longum* subsp. *longum* CECT 4503, *Bacteroides fragilis* DSMZ 2451; *Clostridium coccoides* DSMZ 933; *C. leptum* DSMZ 935; *Lactobacillus casei* ATCC 393; and *E. coli* CECT 4558.

### Statistical analyses

Statistical analyses were performed using SPSS v.15 software (SPSS Inc., Chicago, IL, USA). For normally distributed data ANOVA and the Student *t* test were applied and, for non-normally distributed data, the Mann-Whitney *U* test was used. Statistical significance was established at *P*<0.05 for all comparisons.

## Results

### Body weight and morphometric analyses of jejunal sections

**Figure 1:**
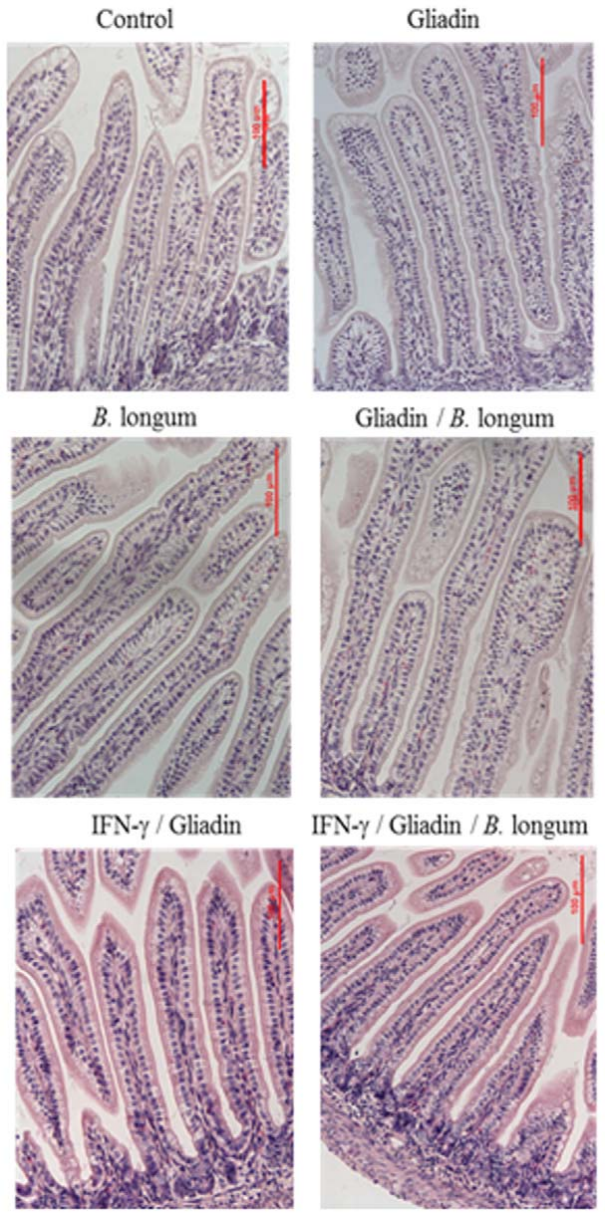
Histology of jejunal tissue sections of different experimental rat groups, stained with hematoxylin-eosin showing the different degree of cellular infiltration in the lamina propria of animals sensitized or not with IFN-γ and fed gliadins and *B. longum* CECT 7347. Scale bar, 100 µm.

Significant (*P*>0.05) differences in body weight were not detected among the different experimental animal groups (*data not shown*). The animals sensitised with interferon (IFN)-γ and fed gliadin were the only ones that presented signs of diarrhoea. The animal group that was diagnosed with diarrhoea had faecal spots around the anal area and the colon contents recovered after the sacrifice had watery consistency. Morphometric analyses of jejunal tissue sections revealed that animals fed with gliadin alone did not exhibit significant alterations compared to controls, except for decreased (*P* = 0.014) enterocyte height (**Table 1**), but this alteration was restored by simultaneous administration of *B. longum* CECT 7347. In animals sensitised with interferon (IFN)-γ and fed gliadin, there was a significant decrease in villi width (*P* = 0.048) and enterocyte height (*P* = 0.033) and an increase in enterocyte numbers (*P* = 0.001) in the apical part of jejunal sections and also higher (*P* = 0.001) infiltration of cells in the lamina propria (**Figure 1**) compared to the controls. However, these changes were not observed in animals only sensitized with IFN-γ but not fed gliadin. *B. longum* CECT 7347 administration increased villi width (*P* = 0.004) and enterocyte height (*P* = 0.005), partially restoring the alterations detected in animals sensitised with IFN-γ and fed gliadin but did not decrease the cellular infiltration observed in the IFN/gliadin group. Feeding of *B. longum* CECT 7347 alone or together with gliadin increased (*P* = 0.016 and *P* = 0.020, respectively) villi length when compared to control animals.

**Table 1:** Morphometric evaluation of jejunal sections.

Treatmentl		Control	Gliadin	*B. longum*	Gliadin/*B. longum*	IFN-γ	IFN-γ/Gliadin	IFN-γ/Gliadin/*B. longum*
Villi	width (µm)	46.15±7.56^a^	42.65±6.39	57.10±6.23	57.53±8.36	48.57±6.35	38.27±8.46^ab^	50.46±2.61^ab^
	length (µm)	193.37±15.53^c^^, ^ d	156.68±29.74	255.83±31.57^d^	247.44±51.74^c^	187.68±26.10	165.88±36.62	196.74±14.00
Infiltrated cells^1^		7.75±1.25^e^^,^ f ^,^ g	8.80±1.64	8.12±0.80	10.24±1.10^e^	6.85±2.12^h^	11.25±0.96^f^^, ^ h	10.52±1.57^g^
Enterocytes	height (µm)	7.04±1.66^i^^,^ j	4.29±1.24^i^^, ^ k	8.32±1.58	7.71±1.01^k^	7.68±1.92	4.79±1.03^j^^, ^ l	7.22±0.96^l^
	counts^2^	4.80±0.80^m,n^	6.83±0.75	5.09±0.62	6.75±1.22	5.12±097	8.20±0.80^m^	8.01±0.35^n^

The results are expressed as mean ± standard deviation of 20 independent microscopic fields of each animal (n = 6).

a-l Superscript letters in the same row indicate statistically significant (*P*<0.05) differences between the pair of samples that has the same letter as determined applying the Student *t* test (*).

1 Number of cells in a surface of 20 µm^2^ at the lamina propria;

2 Number of enterocytes a long 20 µm at the luminal side of the intestinal epithelia.

* *P* values: a, 0.050; b, 0.004; c, 0.020; d, 0.016; e, 0.004; f, 0.001; g, 0.007; h, 0.043; i, 0.014; j, 0.033; k, 0.005; l, 0.005; m, 0.001; n, 0.001.

### NFκB mRNA expression analysis

**Figure 2:**
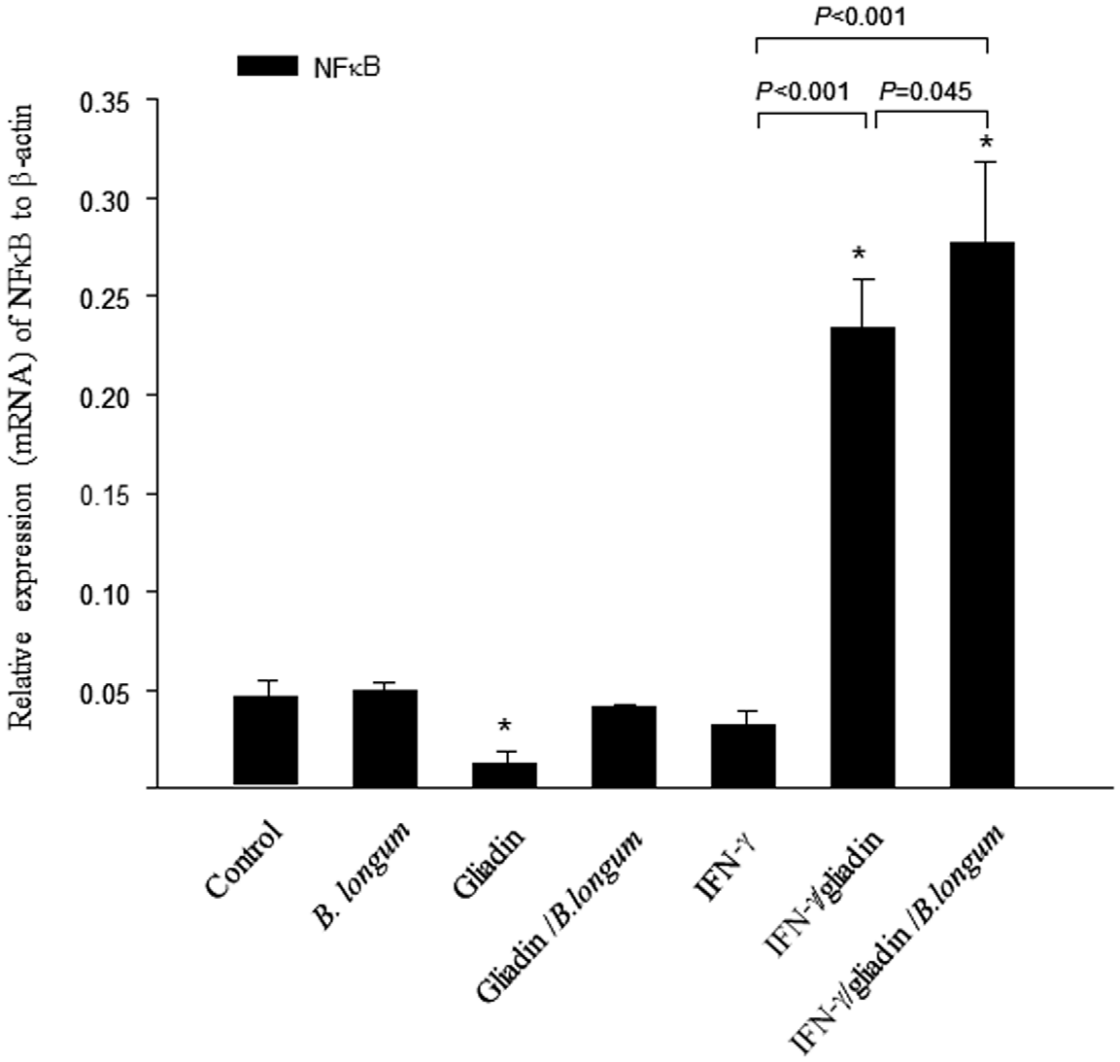
Expression (mRNA) of nuclear factor kappa B (NFκB) in jejunal sections of rats after different treatments. The results are expressed as mean ± standard deviation (n = 6). *Indicates statistically significant (*P*<0.05) differences relative to the controls; bars indicate significant differences between the specific pair comparisons as determined by applying the Student *t* test.

Gliadin feeding significantly (*P* = 0.013) reduced NFκB mRNA expression, while the simultaneous administration of *B. longum* CECT 7347 restored its levels, reaching similar values as those of controls (**Figure 2**). In animals sensitised with IFN-γ and fed gliadin, NFκB expression was markedly increased (*P*<0.001) and the simultaneous administration of *B. longum* CECT 7347 produced even higher NFκB (*P* = 0.045) gene expression. Feeding of *B. longum* CECT 7347 alone to weaning animals did not alter the basal expression of this inflammatory marker, indicating that the intestinal inflammatory milieu and the simultaneous presence of other stimuli modify the immune effects of this bacterial strain. Sensitisation with IFN-γ immediately after birth did not exert a significant effect in comparison with controls.

### Cytokine production

**Figure 3:**
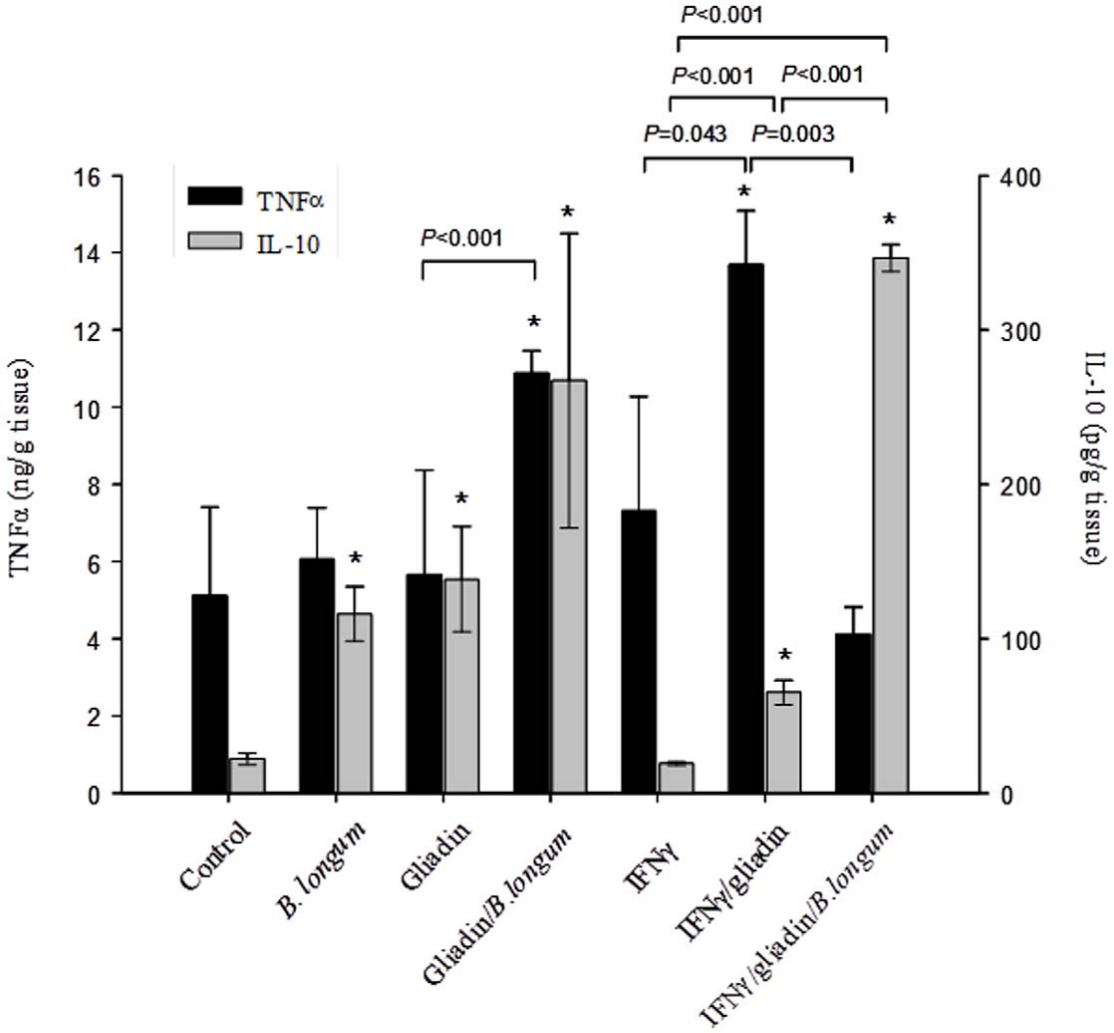
Tumour necrosis factor (TNF)-α and interleukine (IL)-10 production in jejunal tissue sections of rats after different treatments. The results are expressed as mean ± standard deviation (n = 6). *Indicates statistically significant (*P*<0.05) differences compared to the controls; bars indicate significant differences between the specific pair comparisons as determined by applying the Student *t* test.

The cytokine concentrations in jejunal tissue sections from different experimental animal groups quantified by ELISA are shown in **Figure 3**. In tissue samples from animals fed either gliadin or *B. longum* CECT 7347 the concentration of the inflammatory cytokine TNFα was not increased, but that of the anti-inflammatory cytokine IL-10 (*P* = 0.003, *P* = 0.006, respectively) did increase in comparison with controls. The simultaneous administration of both gliadin and *B. longum* CECT 7347 caused a significant increase in TNFα and IL-10 production, in comparison with the control (*P* = 0.012 and *P* = 0.028, respectively) and with the administration of either gliadin (*P*<0.001 and *P* = 0.017, respectively) or *B. longum* CECT 7347 (*P* = 0.029 and *P* = 0.001, respectively) alone. In animals sensitised with IFN-γ and fed gliadin, TNFα concentrations were significantly (*P* = 0.007) increased and, to a lesser extent, also those of IL-10 (*P* = 0.008) in comparison with control animals. This effect could be only attributed to the administration of gliadin in IFN-γ sensitised animals because animals only sensitized with IFN-γ but not fed with gliadin did not exhibit significant increases in cytokine production. The administration of *B. longum* CECT 7347 to the enteropathy model significantly reduced TNFα (*P* = 0.003) and increased IL-10 (*P*<0.001) production, indicating its ability to counteract the inflammatory response in the intestinal mucosa.

### Leukocyte count and lymphocyte phenotyping

The alterations in peripheral leukocyte populations in different animal groups are shown in **Table 2**. Gliadin administration did not cause significant alterations in the lymphocyte population compared to controls, except for an increase in eosinophils (*P* = 0.048). This alteration was restored by simultaneous administration of *B. longum* CECT 7347 (*P* = 0.048). Sensitization of animals with IFN-γ alone did not cause significant changes in leukocyte population, but feeding gliadin to animals sensitised with IFN-γ decreased lymphocyte populations significantly (*P* = 0.028) in comparison to non-sensitised animals that had been fed gliadin. In contrast, monocyte populations significantly increased in sensitized and gliadin-fed animals (*P* = 0.039) and also in those simultaneously fed *B. longum* CECT 7347 (*P* = 0.005) in comparison to controls. The administration of the bifidobacterial strain alone did not significantly modify the leukocyte populations in comparison to controls.

**Table 2:** Leukocyte percentages (%) in peripheral blood of different rat experimental groups.

Cells	Treatment						
	Control	Gliadin	*B. longum*	Gliadin/*B. longum*	IFN-γ	IFN-γ/Gliadin	IFN-γ/Gliadin/*B. longum*
Neutrophil	17.1±3.8	18.6±4.9	20.2±3.9	19.1±1.8	17.2±2.3	22.2±4.0	18.5±5.5
Lymphocyte	73.7±7.1	78.0±5.6^a^	72.5±6.8	73.4±11.1	73.3±1.9	66.8±5.4^a^	69.2±4.8
Monocyte	3.3±0.6^b^^, ^ c	3.6±0.9	2.9±1.1	3.4±1.2	4.1±1.3	6.0±1.9^b^	6.5±1.5^c^
Eosinophilo	4.3±0.7^d^	5.3±0.6^d^^,^ e	5.6±1.3	2.7±1.4^e^	3.3±1.7	4.2±1.2	4.9±1.1
Basophil	1.3±0.4	1.6±0.9	1.1±1.0	1.1±0.2	2.1±1.1	0.8±0.6	0.7±0.5

The results are expressed as mean ± SD (n = 6).

a-e Superscript letters in a same row indicate statistically (*P*<0.05) significant differences between the pair of samples that has the same letter as determined applying the Student *t* test (*).

* *P* values: a, 0.028; b, 0.039; c, 0.005; d, 0.048; e, 0.048.

**Figure 4:**
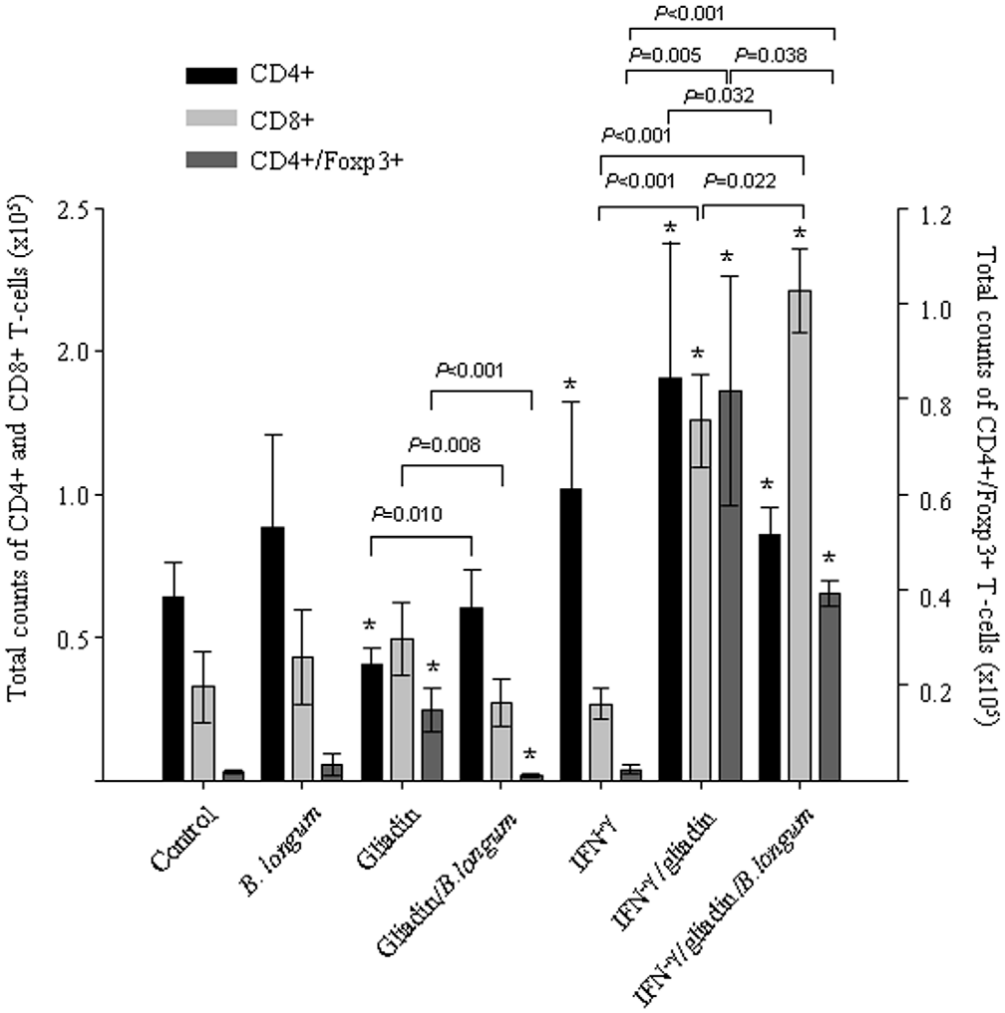
Phenotyping of lymphocyte populations in peripheral blood of rats after different treatments. The results are expressed as mean ± standard deviation (n = 6). *Indicates statistically significant (*P*<0.05) differences relative to the controls; bars indicate significant differences between the specific pair comparisons as determined by applying the Student *t* test.

In rats, leukocyte counts have been reported to be highly variable in the range from 6,000 to 18,000 leukocytes/µl [26], which is in agreement with the values found in our experimental animal groups. The lymphocyte population was identified with the lymphocyte marker CD45 and further T-cell phenotyping analyses were done within this population (**Figure 4**). Feeding gliadin significantly reduced CD4+ lymphocyte numbers (*P* = 0.003) compared to controls, whereas Foxp3+ T cell numbers increased (*P*<0.001). The administration of *B. longum* CECT 7347 together with gliadin exerted the opposite effects in all T-lymphocyte subpopulations, increasing CD4+ cells (*P* = 0.010) and reducing CD8+ (*P* = 0.008) and FoxP3+ T cells (*P*<0.001). Animals sensitized with IFN-γ exhibited significantly (*P* = 0.041) increases in CD4+ cells, but not in CD8+ and CD4+Foxp3 cells. In animals sensitised with IFN-γ and fed gliadin, there was a much more marked increase in all T-lymphocyte subpopulations compared to controls. In this model, the administration of *B. longum* CECT 7347 significantly reduced CD4+ (*P* = 0.032) and Foxp3+ T (*P* = 0.038) cell populations, but increased those of CD8+ cells (*P* = 0.022). The administration of *B. longum* CECT 7347 alone did not significantly affect any of the T cell lymphocyte populations analysed.

### Bacterial translocation and microbiota composition

Translocation of *Bifidobacterium* spp. to liver, spleen and MLN was not detected by plate counting in any of the treated animal groups. RAPD analyses of colonies isolated from selective media for bifidobacteria present in colon samples indicated that the strain administered represented between 75–95% of the total bifidobacteria. The quantitative analyses of specific bacterial groups by real time-PCR also indicated that the administration of the bifidobacterial strain contributed to an increase in the total gene copies of this bacterial group by at least one logarithmic unit (**Table 3**). Neither feeding gliadin alone nor sensitization with IFN-γ alone significantly modified the composition of the microbiota in comparison with controls. In animals sensitised with IFN-γ and fed gliadin, significantly higher gene copy numbers of the *Bacteroides fragilis* group were detected in comparison with controls (*P* = 0.030) and with rats fed gliadin (*P* = 0.020) and gliadin plus *B. longum* CECT 7347 (*P* = 0.049), and with those sensitized with IFN-γ (*P* = 0.025). The administration of *B. longum* CECT 7347 did not restore microbiota alterations in the enteropathy model and only contributed to increasing total *Bifidobacterium* gene copy numbers.

**Table 3:** Microbiota composition of colon content determined by real time PCR and expressed as log copy number of 16S rDNA gene per gram of faeces.

Bacterial group	Control	Gliadin	Gliadin/*B. longum*	*B. longum*	IFN-γ	IFN-γ/gliadin	IFN-γ/gliadin*/B. longum*
*C. coccoides* group	8.99^a^	8.18^b^	6.75^c^	8.37	7.06^d^	10.74	10.72^a^^, ^ b ^, ^ c ^, ^ d
	(5.12–9.80)	(4.30–10.38)	(4.53–9.10)	(5.74–11.14)	(6.94–7.18)	(4.50–11.1)	(8.90–11.09)
*C. leptum* group	5.34	5.28	5.33	5.58	7.16	7.84	8.45
	(5.15–6.65)	(4.04–7.57)	(4.62–5.97)	(4.40–9.33)	(6.07–8.29)	(5.09–9.13)	(4.53–9.12)
*Lactobacillus* group	9.60	9.76	10.01	10.09	9.24	9.47	9.65
	(8.03–10.19)	(9.17–9.98)	(9.14–10.51)	(9.69–10.39)	(8.14–11.10)	(8.50–10.46)	(8.40–10.32)
*Enterobacteriaceae*	9.04	9.43	9.24	9.33	8.27	8.89	9.37
	(8.91–9.84)	(9.12–9.77)	(8.85–9.52)	(9.15–9.42)	(6.58–10.70)	(7.90–9.52)	(7.45–9.91)
*Bifidobacterium*	6.77^e^^, ^ f ^, ^ g	6.17^h^^, ^ i	8.13^e^^, ^ j	8.26^f^^, ^ k	7.81^l^	7.00^h^^, ^ m	10.18^g^^, ^ i ^, ^ j ^, ^ k ^, ^ l ^, ^ m
	(5.89–8.45)	(3.00–9.66)	(7.26–8.61)	(6.97–10.56)	(6.97–9.08)	(4.83–10.57)	(9.29–10.59)
*B. fragilis* group	7.44^n^	7.13^o, p^	5.64^q, r^	7.49	7.15^s^	11.07^n^ ^, o, q, s^	10.80^p, r^
	(3.76–8.69)	(5.93–8.49)	(2.06–8.10)	(3.30–11.41)	(3.60–10.38)	(9.99–11.3)	(7.09–11.39)

Data are expressed as median (range of values) (n = 6).

a-n Superscript letters indicate statistically significant (*P*<0.05) differences between the pair of samples that has the same letter by applying the Mann-Whitney *U* test.

## Discussion

This study reports for the first time the effects of feeding a bifidobacterial strain (*B. longum* CECT 7347) at an early postnatal period on the intestinal mucosal architecture and markers of innate and adaptive immunity in an experimental animal model of gliadin-induced enteropathy.

In previous studies, gliadin administration to inbred rats after weaning [4] or adult mice [6] failed to reproduce the enteropathy, probably due to immunocompetence of the experimental animals and lack of gliadin access beyond the epithelial cell layer. Moreover, feeding gliadin even during the early neonatal period was insufficient to cause mucosal damage and significant alterations in epithelium architecture in agreement with our study [4], [27]. Sensitisation of animals with IFN-γ appeared to be necessary to cause mucosal damage and immunological changes resembling those observed in human CD [9]. IFN-γ administration to weaning animals increases macromolecular transport across Peyer's patches [28], and macrophage priming [29] that favours the full establishment of a jejunal mucosal reaction and the instauration of the enteropathy [9]. However sensitisation with IFN-γ alone did not caused histological alterations in our study, in accordance with previous reports [9]. Thus, the animal model used in the present study approaches an intermediate state between proliferative and destructive phases of CD that, in patients, ultimately lead to complete villous atrophy and disruption of intestinal epithelium integrity, which are major characteristics of fully developed CD [2], [30]. *B. longum* CECT 7347 administration to gliadin-fed animals, sensitised with IFN-γ, partially reduced some of the alterations in jejunal architecture caused by the triggers of the disease, which could theoretically contribute to improving the gut barrier function and preventing gliadin translocation to the submucosa. Similarly, administration of *Lactobacillus casei* ATCC 9595 restored the intestinal damage caused by gliadin in HLA-DQ8 transgenic mice treated with indomethacin [27].

In CD patients, gliadin acts as potent inducer of inflammatory gene expression and cytokine production, including TNFα and NFκB activation [31]–[33]. In this study, NFκB expression (mRNA) was increased in animals sensitised with IFN-γ and fed gliadin, in agreement with the NFκB activation found in the intestinal mucosa of CD patients [32]. NFκB activation has been identified as the mechanism by which gliadin mediates TNFα production in enterocytes [17] and human monocytes [33]. Accordingly, TNFα production was also significantly increased in animals sensitised with IFN-γ and fed gliadin. However, the administration of gliadin alone neither induced NFκB expression nor TNFα production in comparison with controls. It is possible that gliadin administered alone stimulates a regulatory response reflected in the down-regulation of NFκB mRNA expression and the increased IL-10 production leading to tolerance in these animals, which are not genetically predisposed to the disease. In fact, previous authors also demonstrated that IFN-γ administered intraperitoneally was necessary to induce the disease together with oral administration of gliadin [9]. In contrast, the simultaneous administration of *B. longum* CECT 7347 and gliadin increased NFκB mRNA expression and cytokine production in comparison with the group only fed gliadin. This could be due to additional interactions of bacterial components with Toll-like receptors (TLRs) that upon-ligand binding can also activate the NFkB pathway and cytokine production. Therefore, the results indicate that this bacterial strain caused certain immune activation in the simultaneous presence of gliadin; however, these effects were not significant in comparison with the control group except for cytokine production and were not translated in other pathologic signs. Notably, the administration of *B. longum* CECT 7347 to animals sensitised with IFN-γ and fed gliadin reduced TNFα production and increased IL-10 production and NFκB expression, triggering an anti-inflammatory and regulatory response. Interestingly, the two treatments IFN-γ sensitization plus gliadin feeding and IFN-γ sensitization plus gliadin and *B. longum* feeding activated NFkB mRNA expression, but the final effects on cytokine production markedly differed. The effects on these inflammatory markers on our enteropathy animal model are in agreement with the inflammatory role of gliadins in CD patients and enterocytes previously reported [15], [32]. However, an increase in NFκB mRNA expression does not always lead to an inflammatory response because this pathway is regulated at different stages and by diverse mechanisms controlling, for instance, the ubiquitination of the inhibitor IkB, which promotes the translocation of the heterodimer p50/p65 to the nucleus and the final induction of inflammatory cytokines such as TNFα [34]. In this context, it has been reported that some commensal bacteria can induce transient activation or inhibition of the NFκB signalling pathway at different steps that contribute to attenuating and regulating the pro-inflammatory responses. For example *B. thetaiotaomicron* acts downstream NFkB activation by promoting nuclear export of NF-κB subunit relA in complex with PPAR-γ [35], while other commensal bacteria block NFκB at more proximal steps, inhibiting ubiquitination and proteolytic inactivation of the endogenous NF-κB inhibitor IκB [36]. Therefore, it is possible that the *B. longum* strain used in this study only causes a transient activation of NF-kB mRNA expression without enhancing the final production of inflammatory mediators such as TNF-α in the enteropathy animal model.

In addition, our results evidence significant differences between the immunomodulatory properties of *B. longum* CECT 7347 and *L. casei* ATCC 9595, since this latter strain was unable to rescue IL-10 production in the enteropathy model of HLA-DQ8 transgenic mice [27]. IL-10 production was also stimulated by the administration of *B. longum* CECT 7347 alone in control mice but not that of TNF-α, which is an additional indication of the anti-inflammatory properties of this strain also in the absence of other stimuli such as gliadin or an inflammatory condition. IL-10 seems to be indispensable for the induction of oral tolerance to dietary antigens, the inhibition of chemokine production and the antigen-presenting capacity of monocytes and macrophages, and induction of the production of soluble antagonists of pro-inflammatory cytokines such as IL-1 and TNFα [37].

Leukocyte counts and phenotyping analyses of T-cell subsets in peripheral blood support that IFN-γ sensitisation of weaning animals is effective in stimulating a T cell-mediated response to orally administered gliadin antigens, partially mimicking the effect in humans. Monocyte numbers were significantly increased in animals sensitised with IFN-γ and fed gliadin, which suggests a response to inflammatory signals that could not be significantly reduced by *B. longum* CECT 7347. The data from lymphocyte phenotyping indicated that gliadin alone reduces CD4+ T cells and increases CD4+/Foxp3+ T cells, suggesting a regulatory response in agreement with previous data. Although these changes were reversed by *B. longum* CECT 7347 administration, indicating that the bacterium can induce certain immune activation in an opposite direction, these effects were not significant in comparison with controls. Our study also demonstrated that IFN-γ sensitisation, prior to gliadin administration, was necessary to induce an enteropathy mediated by CD4+ T cells, while IFN-γ sensitisation alone did not cause significant changes in lymphocyte subpopulations. In the enteropathy model, the changes in CD4+ T cells were also accompanied by an increase in CD4+/Foxp3+ (Tregs) cells, which suggests the development of a counter-regulatory response, as previously reported [7], [11]. The increased Treg cell numbers is concordant with the increased percentages of circulating regulatory CD4+CD25+Foxp3+ T cells found in untreated, compared to treated (gluten-free diet), CD patients [12], [13]. In this study, feeding of *B. longum* CECT 7347 significantly decreased CD4+ and CD4+/Foxp3 Tregs cells in animals sensitised with IFN-γ, indicating its ability to counteract the Th1-type inflammatory response triggered by gliadin. However, a recent study has demonstrated that *L. casei* ATCC 9595 administration was unable to significantly modulate CD25+ T cell populations in HLA-DQ8 transgenic mice that had been fed gliadins [27]. *B. longum* CECT 7347 also induced CD8+ T cells in this model of enteropathy in agreement with the microbiota-mediated increase in CD8+ lymphocytes previously reported [4]; the role of which in this disease remains to be determined.

Microbiota composition of animals sensitised with IFN-γ and fed gliadins showed increased gene copy numbers of the *Bacteroides fragilis* group in comparison with control animals; these differences were not associated with gliadin intake and, therefore, could be due to the induced alterations in intestinal epithelium architecture and the underlying inflammation in the enteropathy model [30], [31]. This alteration resembles that found in paediatric CD patients that showed increased bacteroides numbers in faeces and duodenal biopsies, but not solely associated with the inflammatory phase of the disease [38], [39]. Bifidobacteria and lactobacilli numbers were not related to gliadin intake or the induced inflammation, in contrast to human data [38], [39]. As expected, administration of *B. longum* CECT 7347 led to increased gene copy numbers of total bifidobacteria in the colon, which could be responsible for the biological effects detected on the mucosa and inflammatory markers under gliadin or IFN-γ and gliadin administration. Studies *in vitro* and *in situ* have reported positive effects of different *Bifidobacterium* strains in the context of CD, including the reduction of gliadin toxicity [17], [40] and inflammatory potential of gliadin peptides on intestinal cells [17]. This study has demonstrated the protective effects of *B. longum* CECT 7347 against the aberrant gliadin response *in vivo*, by reducing inflammatory cytokine production and increasing regulatory cytokine production (IL-10) in the jejunal mucosa, and reduced activation of CD4+ T cells. However, the limitations of the animal model used to fully reproduce the fundamental features of the disease makes necessary to consider the results reported with caution and, undoubtedly, studies in humans will be necessary to really prove beneficial effects of this bacterium on the disease.

## References

[1] Wieser H, Koehler P (2008). The biochemical basis of celiac disease.. Cereal Chem.

[2] Schuppan D, Junker Y, Barisani D (2009). Celiac Disease: From pathogenesis to novel therapies.. Gastroenterology.

[3] Troncone R, Ferguson A (1991). Animal model of gluten induced enteropathy in mice.. Gut.

[4] Stěpanková R, Tlaskalová H, Šinkora J, Jodl J, Frič P (1996). Changes in jejunal mucosa after long-term feeding of germfree rats with gluten.. Scand J Gastroenterol.

[5] Kozakova H, Stěpanková R, Tučková L, Šinkora M, Jelínková L (2000). Humoral and Cellular immune responses in gluten-treated suckling or hand fed rats.. Physiol Res.

[6] Tlaskalová-Hogenová H, Stpánková R, Farré M, Funda DP, Reháková Z (1997). Autoimmune reactions induced by gliadin feeding in germ-free AVN rats and athymic nude mice. Animal models for celiac disease.. Ann N Y Acad Sci.

[7] Black KE, Murray JA, David CS (2002). HLA-DQ determines the response to exogenous wheat proteins: a model of gluten sensitivity in transgenic knockout mice.. J Immunol.

[8] Verdu EF, Huang X, Natividad J, Lu J, Blennerhassett PA (2008). Gliadin dependent neuromuscular and epithelial secretory responses in gluten-sensitive HLA-DQ8 transgenic mice.. Am J Physiol Gastrointest Liver Physiol.

[9] Stepánková R, Kofronová O, Tucková L, Kozáková H, Cebra JJ (2003). Experimentally induced gluten enteropathy and protective effect of epidermal growth factor in artificially fed neonatal rats.. J Pediatr Gastroenterol Nutr.

[10] Westendorf AM, Fleissner D, Deppenmeier S, Gruber AD, Bruder D (2006). Autoimmune-mediated intestinal inflammation-impact and regulation of antigen-specific CD8+ T cells.. Gastroenterology.

[11] D'arienzo R, Stefanile R, Maurano F, Luongo D, Bergamo P (2009). A deregulated immune response to gliadin causes a decreased villus height in DQ8 transgenic mice.. Eur J Immunol.

[12] Kivling A, Nilsson L, Fälth-Magnusson K, Söllvander S, Johanson C (2008). Diverse Foxp3 expression in Children with Type I Diabetes and Celiac Disease.. Ann NY Acad Sci.

[13] Frisullo G, Nociti V, Iorio R, Patanella AK, Marti A (2009). Increased CD4+CD25+Foxp3+ T cells in peripheral blood of celiac disease patients: correlation with dietary treatment. Hum Immunol.. 2009.

[14] Terrazzano G, Sica M, Gianfrani C, Mazzarella G, Maurano F (2007). Gliadin regulates the NK-dendritic cell cross-talk by HLA-E surface stabilization.. J Immunol.

[15] Hoffman RA (2000). Intraepithelial lymphocytes coinduce nitric oxide synthase in intestinal epithelial cells.. Am J Physiol: Gastointest Liver Physiol.

[16] Collado MC, Donat E, Ribes-Koninckx C, Calabuig M, Sanz Y (2009). Specific duodenal and faecal bacterial groups associated with paediatric coeliac disease.. J Clin Pathol.

[17] Laparra JM, Sanz Y (2010). Bifidobacteria inhibit the inflammatory response induced by gliadin in intestinal epithelial cells via modification of toxic peptide generation during digestion.. J Cell Biochem.

[18] Medina M, De Palma G, Ribes-Koninckx C, Calabuig M, Sanz Y (2008). Bifidobacterium strains suppress in vitro the pro-inflammatory milieu triggered by the large intestinal microbiota of coeliac patients.. J Inflamm (Lond).

[19] Livak KJ, Schmittgen TD (2001). Amalysis of Relative Gene Expression Data Using Real-Time Quantitative PCR and the 2^−ΔΔC^
_T_ Method.. Methods.

[20] Akopyanz N, Bukanov NO, Westblom TU, Kresovich S, Berg DE (1992). DNA diversity among clinical isolates of Helicobacter pylori detected by PCR-based RAPD fingerprinting).. Nucleic Acids Res.

[21] Matsuki T, Watanabe K, Fujimoto J, Miyamoto Y, Takada T (2002). Development of 16S rRNA-gene-targeted group-specific primers for the detection and identification of predominant bacteria in human feces.. Appl Environ Microbiol.

[22] Malinen E, Kassinen A, Rinttilä T, Palva A (2003). Comparison of real-time PCR with SYBR Green I or 5′-nuclease assays and dot-blot hybridization with rDNA-targeted oligonucleotide probes in quantification of selected faecal bacteria.. Microbiology.

[23] Yu Y, Lee C, Kim J, Hwang S (2005). Group-specific primer and probe sets to detect methanogenic communities using quantitative real-time polymerase chain reaction.. Biotechnol Bioeng.

[24] Birnboim HC, Doly J (1979). A rapid alkaline extraction procedure for screening recombinant plasmid DNA.. Nucleic Acids Res.

[25] Whelan JA, Russell NB, Whelan MA (2003). A method for the absolute quantification of cDNA using real-time PCR.. J Immunol Methods.

[26] Ringler, Dabich (1979).

[27] D'Arienzo R, Stefanile R, Maurano F, Mazzarella G, Ricca E (2010). Immunomodulatory effects of *Lactobacillus casei* administration in a mouse model of gliadin-sensitive enteropathy.. Scand J Immunol.

[28] Sütas Y, Aution S, Rantala I, Isolauri E (1997). IFN gamma enhances macromolecular transport across Peyer's patches in suckling rats: Implications for natural immune responses to dietary antigens early in life.. J Pediatr Gastroenterol Nutr.

[29] Williams JG, Jurkovich GJ, Hahnel GB, Maier RV (1992). Macrophage priming by interferon γ: a selective process with potencially harmful effects.. J Leukoc Biol.

[30] Clemente MG, De Virgilis S, Kang JS, Macatagney R, Musu MP (2003). Early effects of gliadin on enterocyte intracellular signaling involved in intestinal barrier function.. Gut.

[31] Thomas K, Sapone A, Fasano A, Vogel SN (2006). Gliadin stimulation of murine macrophage inflammatory gene expression and intestinal permeability are MyD88-dependent: Role of the innate immune response in celiac disease.. J Immunol.

[32] Maiuri MC, De Stefano D, Mele G, Fecarotta S, Greco L (2003). Nuclear factor κB is activated in samll intestinal mucosa of celiac patients.. J Mol Med.

[33] Jelínková L, Tuckova L, Cinová J, Flegelová Z, Tlaskalová-Hogenová H (2004). Gliadin stimulates human monocytes to production of IL-8 and TNFα through a mechanism involving NFκB.. FEBS Letters.

[34] Viatour P, Merville MP, Bours V, Chariot A (2005). Phosphorylation of NF-κB and IκB proteins: implications in cancer and inflammation.. Trends Biochem Sci.

[35] Kelly D, Campbell JI, King TP, Grant G, Jansson EA, Coutts AG, Pettersson S, Conway S (2004). Commensal anaerobic gut bacteria attenuate inflammation by regulating nuclear-cytoplasmic shuttling of PPAR-gamma and RelA. Nat.. Immunol.

[36] Neish AS, Gewirtz AT, Zeng H, Young AN, Hobert ME (2000). Prokaryotic regulation of epithelial responses by inhibition of IkappaB-alpha ubiquitination.. Science.

[37] Izcue A, Coombes JL, Powrie F (2009). Regulatory Lymphocyes and Intestinal Inflammation.. Annu Rev Immunol.

[38] Nadal I, Donat E, Ribes-Koninckx C, Calabuig M, Sanz Y (2007). Imbalance in the composition of the duodenal microbiota of children with celiac disease.. J Med Microbiol.

[39] Collado MC, Donat E, Ribes-Koninckx C, Calabuig M, Sanz Y (2008). Imbalances in faecal and duodenal *Bifidobacterium* species composition in active and non-active coeliac disease.. BMC Microbiol.

[40] De Palma G, Cinova J, Stepankova R, Tuckova L, Sanz Y (2010). Pivotal Advance: Bifidobacteria and Gram-negative bacteria differentially influence immune responses in the proinflammatory milieu of celiac disease.. J Leukoc Biol.

